# Awareness and attitudes regarding artificial intelligence in medical imaging: student perspectives from five Ghanaian universities

**DOI:** 10.3389/fmed.2026.1903859

**Published:** 2026-09-24

**Authors:** Issah Zabsonre Alhassan, Abdul-Salam Issahaku, Fatahia Alidu, Mohammed Rashad Suhuyini Abdul-Bach

**Affiliations:** 1 Department of Medical Imaging, School of Allied Health Sciences, University for Development Studies, Tamale, Ghana; 2 Department of Applied Physics, School of Physical Sciences, University of Technology and Applied Sciences, Navrongo, Ghana; 3 Department of Medical Physics, School of Nuclear and Allied Sciences, University of Ghana, Accra, Ghana

**Keywords:** artificial intelligence, curriculum, Ghana, medical imaging, radiography, student attitudes

## Abstract

**Background:**

Artificial intelligence (AI) is influencing diagnostic medical imaging practice and education. Despite this, little is known about AI use in education and practice in sub-Saharan Africa. As a first step towards informing future curriculum development, we assessed AI awareness, self-reported familiarity, and attitudes among Ghanaian diagnostic medical imaging students.

**Methods:**

This cross-sectional institution-based study in Ghana used a self-administered 21-item questionnaire to collect data from diagnostic medical imaging students (*N* = 296) of the University of Cape Coast (UCC), University for Development Studies (UDS), Kwame Nkrumah University of Science and Technology (KNUST), University of Health and Allied Sciences (UHAS), and University of Ghana (UG) from June to August 2024. Binary logistic regression was used to examine factors associated with support for including AI as a core component of the medical imaging curriculum.

**Results:**

Nearly all respondents (95.3%) were aware of AI, and 85.8% had ever used an AI product. Most participants also considered AI an emerging trend in medical imaging (85.8%) and agreed that it has a net positive impact on medical imaging (76.0%). Seventy-six percent of participants supported the inclusion of AI in core medical imaging courses. In multivariable logistic regression, male gender (OR = 1.94, 95% CI: 1.07–3.51, *p* = 0.030) and agreement that AI will positively affect medical imaging (OR = 2.99, 95% CI: 1.56–5.73, *p* = 0.001) were associated with support for AI curriculum integration after adjustment. UDS enrolment showed a weak association with curriculum endorsement (OR = 2.45, 95% CI: 1.00–5.97, *p* = 0.049). Academic level and perceived job threat were not independently associated with endorsement of AI curriculum inclusion.

**Conclusion:**

Ghanaian diagnostic medical imaging students demonstrated high awareness of AI, substantial self-reported exposure to AI-enabled products or services, and generally positive attitudes toward its potential applications in medical imaging. A positive perception of AI's overall impact was associated with greater support for formal AI education. These findings indicate strong student receptiveness to AI education but should not be interpreted as evidence of objective AI knowledge or competency.

## 1 Introduction

Artificial intelligence (AI) has evolved from a theoretical, academic area of computer science to a commercial reality in diagnostic radiology and clinical medical imaging (1). Deep learning algorithms, particularly convolutional neural networks (CNNs), trained on large datasets have demonstrated diagnostic performance comparable to that of clinical radiologists for selected applications, including pulmonary nodule detection on chest radiographs and automated lesion classification (2). In high-income healthcare systems, the development of these AI tools is rapidly being translated into clinical practice to improve clinical workflows, shorten diagnostic turnaround time, and reduce observer fatigue, thus requiring a global review of the training and expertise required of professional radiographers, sonographers, and imaging clinicians (3).

Alongside the growth of clinical AI applications, increasing attention has been directed toward how healthcare students understand and perceive AI and how AI-related concepts should be incorporated into professional curricula, including in low- and middle-income countries (LMICs) and sub-Saharan Africa (4–6). Ghana has established undergraduate professional education in diagnostic medical imaging across five public universities: the University of Cape Coast (UCC), University for Development Studies (UDS), Kwame Nkrumah University of Science and Technology (KNUST), University of Health and Allied Sciences (UHAS), and University of Ghana (UG). These accredited programmes contribute to the training of Ghana's diagnostic medical imaging workforce, which supports a healthcare system in which projection radiography and ultrasonography remain important first-line diagnostic modalities․

Although previous Ghanaian studies have examined awareness and perceptions of AI among diagnostic medical imaging students and practicing imaging professionals, important questions remain regarding students' acceptance of formal AI education. Ampofo et al. (8) provided useful descriptive evidence on AI knowledge and awareness among diagnostic imaging students in Ghana. However, descriptive estimates alone do not establish whether support for formal AI education varies according to students' sociodemographic characteristics, institutional affiliation, clinical exposure, or perceptions of the professional implications of AI. Evidence on these associations is relevant as medical imaging programmes consider whether and how AI should be incorporated into undergraduate training.

The present study therefore extends the existing Ghanaian literature by examining students from all five accredited public universities offering undergraduate diagnostic medical imaging programmes and by evaluating factors associated with support for incorporating AI into the formal medical imaging curriculum. Rather than treating awareness and curriculum acceptance as equivalent, the study distinguishes students' familiarity and perceptions of AI from their explicit endorsement of its inclusion in core medical imaging education.

Accordingly, the study aimed to: (1) assess AI awareness, self-reported familiarity, and attitudes toward AI among undergraduate diagnostic medical imaging students across the five participating universities in Ghana; and (2) identify sociodemographic, institutional, and attitudinal factors associated with students' support for incorporating AI into the formal medical imaging curriculum.

## 2 Materials and Methods

### 2.1 Study design and setting

This was a cross-sectional survey conducted between June 2024 and August 2024. Participants were recruited from five universities in Ghana with accredited undergraduate programmes in diagnostic medical imaging. These are the University of Cape Coast (UCC), University for Development Studies (UDS), Kwame Nkrumah University of Science and Technology (KNUST), University of Health and Allied Sciences (UHAS), and University of Ghana (UG).

### 2.2 Sample size determination and sampling procedure

The total number of eligible diagnostic medical imaging students from the five universities was 1,053. The minimum sample size was calculated using Yamane's formula, *n* = (*N*/[1 + *N*(*e*^2^)]). N is the population size, and e is the level of precision (7). Using a population size (*N*) of 1053 and a precision level (*e*) of 0.05, the minimum required sample size was 290 students. The five participating universities were treated as institutional strata for proportional allocation of the target sample. The calculated sample was proportionately distributed according to the eligible student population within each institution, resulting in target samples of 87 for UCC, 65 for UDS, 65 for KNUST, 29 for UG, and 44 for UHAS. Within each institutional stratum, however, participants were recruited using convenience sampling until the allocated quota was reached. The questionnaire was distributed electronically through class WhatsApp platforms and institutional student networks. Because the survey link was disseminated through class WhatsApp platforms and institutional student networks without individualized invitation tracking, the number of eligible students who received or viewed the survey invitation could not be determined. Consequently, a conventional survey response rate could not be calculated. The 296 responses analyzed represented 28.1% of the total eligible student population of 1,053; however, this proportion should not be interpreted as a conventional response rate. Therefore, although proportional institutional quotas were used to improve representation across the five universities, selection of individual participants within each institution was non-probabilistic.

### 2.3 Data collection instrument

Data were collected using a self-administered 21-item questionnaire developed for the study. The questionnaire was informed by previous literature examining knowledge, awareness, and perceptions of AI among diagnostic medical imaging students and professionals (8). Before the main data collection, the questionnaire was pilot-tested among diagnostic medical imaging students who were not included in the final study sample to assess the clarity and comprehensibility of the items. Feedback obtained during the pilot testing was used to refine the wording of the questionnaire before its final administration. The instrument was also reviewed by experts in medical imaging for relevance, clarity, and content coverage.

The questionnaire consisted of five sections. Part 1 collected sociodemographic information, including age group, gender, institution, academic level, and imaging specialty. Part 2 contained five Yes/No/Not sure items assessing general awareness of AI, self-reported AI use, and perceptions of its potential effects. Part 3 contained five Agree/Disagree/Neutral items assessing awareness and perceptions of AI in diagnostic medical imaging. Part 4 contained seven Agree/Disagree/Neutral items examining perceptions of the potential implications of AI for medical imaging practice. Part 5 contained four Yes/No/Not sure items addressing academic curriculum and clinical experience.

As a *post hoc* assessment of internal consistency, Cronbach's alpha was calculated for the multi-item Likert-type domains. Cronbach's alpha was 0.46 for the awareness domain (Q6–Q10) and 0.57 for the perspective domain (Q11–Q17), indicating limited internal consistency. Given these low reliability estimates, the domains were not treated as unified constructs and were not used to generate composite scale scores. The items addressed conceptually heterogeneous aspects of AI, including perceived benefits, professional concerns, implementation barriers, and anticipated effects on medical imaging practice. Accordingly, all subsequent analyses were conducted using individual questionnaire items rather than composite awareness or perspective scores. The attitudinal component of the questionnaire consisted predominantly of closed-ended items in which respondents indicated their level of agreement with prespecified propositions. Several items were declaratively worded and addressed potential professional or implementation consequences of AI, including job security, professional displacement, professional knowledge, workload, and implementation cost. Responses to these items were therefore interpreted as endorsement or non-endorsement of the propositions presented rather than as spontaneously generated concerns.

### 2.4 Outcome variable

The primary outcome was derived from item 20, “Do you think that AI should be taught in core courses of medical imaging?” Responses of “Yes” were coded as 1 and represented affirmative endorsement of AI curriculum integration. “No” and “Not sure” responses were combined and coded as 0 because neither represented explicit endorsement of curriculum inclusion. This binary classification was selected because the primary analytical question concerned factors associated with affirmative support for formal AI education. However, because uncertainty may be conceptually different from disagreement, a sensitivity analysis was conducted in which “Not sure” responses were excluded, and the analysis was repeated comparing “Yes” with “No” responses.

### 2.5 Statistical analysis

Descriptive statistics were computed for all variables, reported as frequencies and percentages. Chi-square tests assessed bivariate associations between the primary outcome and sociodemographic variables and key attitudinal items. Binary logistic regression was used to examine factors independently associated with support for including AI in core medical imaging courses, with gender, academic level, institution, perceived AI job threat (Q5), belief in AI displacement of radiographers (Q8), belief that AI cost would hinder implementation in Ghana (Q17), perceived increase in AI knowledge through clinical practice (Q18), and overall positive view of AI in medical imaging (Q10) entered as covariates. These predictors were selected based on their conceptual relevance and previous literature concerning sociodemographic, institutional, professional, implementation, and attitudinal factors potentially associated with acceptance of AI education, rather than solely based on statistical significance in bivariate analyses. Multicollinearity among the predictors was assessed using variance inflation factors (VIFs) and tolerance values. VIFs ranged from 1.06 to 1.78 and tolerance values from 0.56 to 0.95, indicating no evidence of problematic multicollinearity. Missing item-level responses were minimal and were not imputed. Descriptive and bivariate analyses were conducted using available responses for the variables included in each analysis. For analyses involving gender, respondents who selected “Prefer not to say” were retained in the descriptive analysis but excluded from the inferential gender comparison because of the small size of this category. The multivariable logistic regression therefore included respondents with an available outcome who reported male or female gender (*N* = 290). Female gender, Level 400, and KNUST were specified as the reference categories for gender, academic level, and institution, respectively. Adjusted odds ratios (ORs) with 95% confidence intervals (CIs) were reported. Model performance was assessed using McFadden's R^2^, while model calibration was evaluated using the Hosmer–Lemeshow goodness-of-fit test. A non-significant Hosmer-Lemeshow test (*p* > 0.05) was considered indicative of no evidence of poor model calibration. Analysis was conducted using Python (SciPy, scikit-learn). Significance was set at *p* < 0.05.

### 2.6 Ethical considerations

This study was approved by the Institutional Review Board of the University for Development Studies (Approval No.: UDSIRB/164/24). Participation was voluntary. Electronic informed consent was obtained before the questionnaire was filled in. No identifiers were collected. The preamble to the questionnaire stated that responses would be treated in confidence and that there was no risk involved in participating.

## 3 Results

### 3.1 Sociodemographic characteristics

A total of 296 students were included in the final analysis. Of the 296 participants, 76.0% were aged 18–24 years and 65.2% were male. UCC accounted for the largest proportion of respondents (30.1%), followed by UDS (22.0%), KNUST (22.0%), UHAS (15.2%), and UG (9.5%). Level 400 students constituted the largest academic-level group (37.8%), while radiography was the most frequently reported specialty (64.2%). Participant characteristics are presented in Table 1.

**Table 1:** Sociodemographic characteristics of diagnostic medical imaging students, Ghana (*N* = 296).

Characteristic	*N*	% (of *N* = 296)
Age group		
18–24 years	225	76.0
25–30 years	61	20.6
31–35 years	4	1.4
36–40 years	1	0.3
41 years and above	3	1.0
Missing	2	0.7
Gender		
Male	193	65.2
Female	98	33.1
Prefer not to say	5	1.7
Institution		
University of Cape Coast (UCC)	89	30.1
University for Development Studies (UDS)	65	22.0
KNUST	65	22.0
University of Health and Allied Sciences (UHAS)	45	15.2
University of Ghana (UG)	28	9.5
Missing	4	1.4
Academic level		
Level 200	84	28.4
Level 300	95	32.1
Level 400	112	37.8
Missing	5	1.7
Specialty		
Radiography	190	64.2
Sonography	60	20.3
Yet to specialise	44	14.9
Missing	2	0.7

Percentages may not total 100% due to rounding and missing data.

### 3.2 General awareness, self-reported AI use, and perceptions (part 2)

Most respondents (95.3%) reported having heard of AI before the survey, while 91.9% agreed that AI has the potential to affect various industries. Furthermore, 85.1% perceived that AI could improve efficiency and accuracy in tasks typically performed by humans, and 85.8% reported previous use of an AI-enabled product or service. Views regarding employment effects were less consistent, with 53.4% endorsing the proposition that AI poses a threat to job security and 34.8% responding negatively to this proposition. The distributions are presented in Table 2.

**Table 2:** General awareness, self-reported AI use, and perceptions among diagnostic medical imaging students (*N* = 296).

Item	Yes *n* (%)	No *n* (%)	Not sure *n* (%)
Q1. Have you heard about AI before this questionnaire?	282 (95.3)	8 (2.7)	5 (1.7)
Q2. AI has the potential to significantly impact various industries?	272 (91.9)	15 (5.1)	8 (2.7)
Q3. Have you ever used an AI-enabled product or service?	254 (85.8)	29 (9.8)	13 (4.4)
Q4. AI can enhance efficiency and accuracy in human tasks?	252 (85.1)	20 (6.8)	23 (7.8)
Q5. AI poses a threat to job security?	158 (53.4)	103 (34.8)	35 (11.8)

*N* for each item varies slightly due to missing responses (maximum one missing per item).

### 3.3 Awareness and perspectives on AI in medical imaging (parts 3 and 4)

Regarding perceptions of AI in medical imaging, 85.8% of respondents agreed that AI is an emerging trend in the field, while 75.3% regarded AI as part of the future of medical imaging. Views regarding professional displacement were more varied. Overall, 38.2% agreed that AI could eventually displace radiographers, while 45.9% disagreed. In addition, 60.5% agreed that machine-related errors could occur with AI-enabled equipment.

Respondents generally reported favourable perceptions of several potential applications of AI in medical imaging. Large majorities supported reducing radiation dose while preserving image quality (83.8%), improving decision-making (81.4%), enhancing diagnostic accuracy (79.4%), and facilitating access to care in underserved regions (73.3%). Views were more divided on whether AI would result in less knowledge for radiographers (44.9% agree, 40.5% disagree) and whether it would increase workload (31.8% agree, 56.4% disagree). Regarding implementation barriers, 72.3% of respondents agreed that high cost could hinder artificial intelligence implementation in Ghana. The results are shown in Table 3.

**Table 3:** Awareness and perspectives on AI in medical imaging (*N* = 296).

Item	Agree *n* (%)	Disagree *n* (%)	Neutral *n* (%)
PART 3: Awareness about AI in Medical Imaging			
Q6. AI is an emerging trend in medical imaging.	254 (85.8)	15 (5.1)	26 (8.8)
Q7. AI technology is the future of medical imaging.	223 (75.3)	30 (10.1)	38 (12.8)
Q8. AI integration would someday displace you as a radiographer.	113 (38.2)	136 (45.9)	47 (15.9)
Q9. Machine errors are possible with AI-induced equipment.	179 (60.5)	69 (23.3)	47 (15.9)
Q10. AI would have an overall positive impact on medical imaging.	225 (76.0)	29 (9.8)	42 (14.2)
PART 4: Perspective on AI in Medical Imaging			
Q11. AI will reduce radiation dose while maintaining image quality.	248 (83.8)	25 (8.4)	23 (7.8)
Q12. AI integration will result in less knowledge for radiographers.	133 (44.9)	120 (40.5)	41 (13.9)
Q13. AI integration will increase the radiographer's workload.	94 (31.8)	167 (56.4)	34 (11.5)
Q14. AI will increase access to care where radiologists are absent.	217 (73.3)	31 (10.5)	44 (14.9)
Q15. AI has increased accuracy in disease detection and diagnosis.	235 (79.4)	25 (8.4)	33 (11.1)
Q16. AI will improve decision-making on diagnostic results.	241 (81.4)	27 (9.1)	27 (9.1)
Q17. AI implementation in Ghana will be hindered by high cost.	214 (72.3)	34 (11.5)	48 (16.2)

Items from Parts 3 and 4 of the questionnaire. Responses recorded on an Agree/Disagree/Neutral scale.

### 3.4 Academic curriculum and clinical practice (part 5)

Clinical exposure was perceived to increase knowledge of AI in medical imaging by 68.9% of respondents, while 69.6% reported that clinical practice increased their appreciation of AI applications. A clear majority (76.0%) supported the inclusion of AI in core medical imaging courses, and 85.8% expressed an interest in learning about AI and its potential future applications.

### 3.5 Bivariate associations with the primary outcome

Bivariate analysis showed a statistically significant association between gender and support for including AI in core medical imaging courses among respondents who reported male or female gender (*χ*^2^ = 6.80, df = 1*, p* = 0.009), with a greater proportion of male than female students supporting curriculum inclusion. Academic level (*χ*^2^ = 1.36, df = 2, *p* = 0.507) and institution (*χ*^2^ = 2.14, df = 4*, p* = 0.711) were not significantly associated with curriculum endorsement. Among the attitudinal variables, agreement that AI would have an overall positive impact on medical imaging was significantly associated with curriculum endorsement (*χ*^2^ = 16.50, df = 1, *p* < 0.001). Significant associations were also observed for the belief that clinical practice increases AI knowledge (*χ*^2^ = 4.22, df = 1, *p* = 0.040) and agreement that high cost would hinder AI implementation in Ghana (*χ*^2^ = 6.06, df = 1, *p* = 0.014). Full bivariate results are presented in Table 4.

**Table 4:** Bivariate associations between selected variables and support for including AI in core medical imaging courses.

Variable	AI curriculum integration: Yes *n* (%)	AI curriculum integration: No/Not sure *n* (%)	chi-square	df	*p*-value
Gender			6.80	1	0.009
Male	156 (80.8)	37 (19.2)			
Female	65 (67.0)	32 (33.0)			
Prefer not to say	4 (100.0)	0 (0.0)			
Academic level			1.36	2	0.507
Level 200	68 (81.0)	16 (19.0)			
Level 300	70 (75.3)	23 (24.7)			
Level 400	83 (74.1)	29 (25.9)			
Institution			2.14	4	0.711
UCC	66 (74.2)	23 (25.8)			
UDS	53 (81.5)	12 (18.5)			
KNUST	48 (75.0)	16 (25.0)			
UHAS	37 (82.2)	8 (17.8)			
UG	20 (74.1)	7 (25.9)			
AI overall positive impact (Q10)			16.50	1	<0.001
Agree	184 (82.1)	40 (17.9)			
Disagree/Neutral	41 (58.6)	29 (41.4)			
Clinical practice increases AI knowledge (Q18)			4.22	1	0.040
Yes	163 (79.9)	41 (20.1)			
No/Not sure	62 (68.9)	28 (31.1)			
AI cost hindrance, Q17			6.06	1	0.014
Agree	171 (80.3)	42 (19.7)			
Disagree/Neutral	54 (66.7)	27 (33.3)			

Percentages are row percentages. Chi-square tests were performed using the binary outcome variable: Yes vs. No/Not sure. For gender, respondents who selected “Prefer not to say” were retained descriptively but excluded from the inferential comparison because of the small size of this category; the reported gender chi-square statistic therefore represents the male-vs.-female comparison. Cases with missing data for the variables included in each test were excluded from that specific analysis.

### 3.6 Multivariable logistic regression

In the primary adjusted model, male gender and a positive perception of AI's overall impact were associated with curriculum endorsement, although subsequent sensitivity analysis indicated that the gender association was not robust to alternative classification of the outcome. Male students had higher odds of supporting AI curriculum integration than female students (OR = 1.94, 95% CI: 1.07–3.51, *p* = 0.030). Enrolment at UDS was weakly associated with higher odds of curriculum endorsement compared with KNUST (OR = 2.45, 95% CI: 1.00–5.97, *p* = 0.049). Agreement that AI would have an overall positive impact on medical imaging showed the strongest association with curriculum endorsement (OR = 2.99, 95% CI: 1.56–5.73, *p* = 0.001). Academic level, perceived employment threat, perceived displacement, cost concerns, and exposure to clinical practice were not statistically associated with the outcome after adjustment. Among institutions, UDS showed a weak association compared with KNUST, while the estimates for the other institutions did not reach statistical significance. The model fit was modest (McFadden's R-squared = 0.099), while the Hosmer–Lemeshow goodness-of-fit test was non-significant (*χ*^2^ = 7.01, df = 8, *p* = 0.535), indicating no evidence of poor model calibration. Table 5 reports the full regression analysis results.

**Table 5:** Multivariable logistic regression of factors associated with support for including AI in core medical imaging courses (*N* = 290).

Variable	OR	95% CI	*p*-value
Gender (ref: Female)			
Male	1.94	1.07–3.51	0.030
Academic level (ref: Level 400)			
Level 200	1.56	0.74–3.29	0.245
Level 300	1.11	0.56–2.19	0.770
Institution (ref: KNUST)			
UCC	1.56	0.71–3.44	0.271
UDS	2.45	1.00–5.97	0.049
UHAS	2.73	0.99–7.51	0.052
UG	1.15	0.40–3.35	0.794
AI perceived as job threat (Q5)			
Yes (ref: No/Not sure)	1.08	0.59–1.96	0.799
AI will displace radiographers (Q8)			
Agree (ref: Disagree/Neutral)	1.29	0.67–2.47	0.448
AI cost hindrance in Ghana (Q17)			
Agree (ref: Disagree/Neutral)	1.50	0.78–2.90	0.228
Clinical practice increases AI knowledge (Q18)			
Yes (ref: No/Not sure)	1.32	0.70–2.50	0.397
AI overall positive impact (Q10)			
Agree (ref: Disagree/Neutral)	2.99	1.56–5.73	0.001
Model performance: McFadden's *R*^2^ = 0.099. Hosmer–Lemeshow goodness-of-fit test: *χ*^2^ = 7.01, df = 8, *p* = 0.535. *N* = 290.			

OR, odds ratio; CI, confidence interval. Respondents with an available outcome who reported male or female gender were included in the multivariable model (*N* = 290). Respondents who selected “Prefer not to say” for gender were excluded because of the small size of this category. Female gender, Level 400, and KNUST were used as reference categories.

### 3.7 Sensitivity analysis

When respondents who selected “Not sure” for the primary outcome were excluded, 246 participants were included in the sensitivity analysis comparing “Yes” with “No” responses. A positive perception of AI's overall impact remained associated with curriculum endorsement (adjusted OR = 4.03, 95% CI: 1.61–10.06, *p* = 0.003). However, the association with male gender was no longer statistically significant (adjusted OR = 0.96, 95% CI: 0.38–2.40, *p* = 0.930). UDS (adjusted OR = 4.18, 95% CI: 1.20–14.49, *p* = 0.024) and UHAS (adjusted OR = 13.94, 95% CI: 1.59–121.97, *p* = 0.017) showed higher odds of endorsement, although the estimates had wide confidence intervals. These findings indicate that the association with positive perceptions of AI was maintained across outcome definitions, whereas the gender and institution-specific estimates were unstable and should be interpreted cautiously, particularly given the wide confidence intervals for some institutional comparisons.

## 4 Discussion

This multi-institutional quantitative study examined AI awareness, self-reported use, and attitudes among undergraduate diagnostic medical imaging students across all accredited public universities in Ghana. The findings show high baseline awareness of AI, substantial self-reported exposure to AI-enabled tools, and strong support for including AI in medical imaging education. At the same time, substantial proportions of respondents endorsed prespecified propositions concerning job security, potential loss of professional knowledge, and the cost of AI implementation. These findings suggest that future AI curriculum development in Ghana should consider students' receptiveness to AI education alongside their perceptions of the potential professional and practical implications of AI.

Nearly all respondents (95.3%) had heard of AI, while 85.8% reported previous use of an AI-enabled product or service. These findings indicate substantial baseline awareness and self-reported exposure to AI among diagnostic medical imaging students. However, the questionnaire did not distinguish between general-purpose AI applications and AI tools specifically designed for medical imaging. The reported level of AI use should therefore not be interpreted as evidence of clinical AI experience or competency. Nevertheless, the high baseline awareness provides a useful foundation upon which structured AI education in medical imaging could be developed (4, 6).

Although most respondents recognised potential benefits of AI in medical imaging, responses to prespecified propositions concerning its potential professional implications were more mixed. A large proportion of respondents perceived potential benefits of AI in reducing radiation dose while maintaining image quality (83.8%), improving decision-making on diagnostic results (81.4%), and increasing accuracy in disease detection and diagnosis (79.4%) (4). However, 53.4% agreed with the proposition that AI poses a potential threat to job security, while 38.2% considered the displacement of radiographers by AI to be possible. These responses represent endorsement of propositions presented in the questionnaire rather than concerns spontaneously identified by respondents. Similar perceptions have been reported among medical students in the United Kingdom, where Sit et al. found that perceptions of AI influenced students' views about radiology and future career choice (4, 9, 10).

Concerns about the professional implications of emerging technologies have also been reported among healthcare professionals in sub-Saharan Africa. These professional concerns included both potential job displacement and the possible loss of traditional diagnostic knowledge associated with increasing reliance on automated systems. In the present study, 44.9% of respondents agreed with the prespecified proposition that AI integration could result in reduced professional knowledge among radiographers. Concerns regarding the professional implications of increasing automation have also been described in previous research (4). The findings suggest that clinical exposure alone does not explain students' support for AI curriculum integration, and other educational and individual factors may warrant investigation.

In the present study, 72.3% of respondents agreed with the prespecified proposition that high cost could hinder AI implementation in Ghana. Cost and other implementation challenges have similarly been identified in previous Ghanaian and resource-constrained settings (5, 6). Similar concerns have been reported among healthcare professionals in low- and middle-income countries, where limitations in digital infrastructure, picture archiving and communication systems, reliable power supply, and access to advanced image-processing technologies may constrain AI implementation (6, 11). These contextual challenges suggest that AI education in resource-constrained settings should address not only foundational concepts but also the practical requirements, infrastructure limitations, and cost considerations associated with local clinical implementation (6, 12).

The multivariable analysis provides additional insight into factors associated with students' support for integrating AI into the formal medical imaging curriculum. Among the variables included in the model, perceiving AI as having an overall positive impact on medical imaging showed the strongest association with curriculum endorsement [OR = 2.99, 95% CI (1.56, 5.73), *p* = 0.001]. Students with a positive perception of AI therefore had higher odds of supporting its formal inclusion in the curriculum than those who disagreed or were neutral. However, because the study was cross-sectional, the direction of this relationship cannot be established. A favourable perception of AI may contribute to support for formal AI education, but students who already favour AI education may also be more likely to perceive its professional impact positively. Educational programmes should therefore provide balanced exposure to the potential applications, limitations, ethical considerations, and practical implications of AI in medical imaging (13).

Male students had higher odds of endorsing AI curriculum integration than female students [OR = 1.94, 95% CI (1.07, 3.51), *p* = 0.030]. However, this association was not maintained when students who were uncertain about curriculum inclusion were excluded in the sensitivity analysis, further supporting cautious interpretation of the gender finding. The factors underlying the association observed in the primary analysis cannot be established from the present study. Although previous research has reported gender-related differences in attitudes toward and engagement with digital technologies, such differences may reflect several interacting educational, social, and contextual factors rather than gender itself. The present study did not assess digital literacy, previous AI training or exposure, access to computing resources, socioeconomic circumstances, or other factors that could explain the observed association.

The finding should therefore not be interpreted as evidence of differences in technological ability or readiness for AI education between male and female students. Further research incorporating these potential explanatory factors is required to understand the observed gender difference.

Enrolment at UDS was associated with higher odds of endorsing AI curriculum integration compared with KNUST (OR = 2.45, 95% CI: 1.00–5.97, *p* = 0.049). However, this finding should be interpreted cautiously because the confidence interval approached the null value and the overall bivariate association between institution and curriculum endorsement was not statistically significant. Furthermore, the study did not assess institution-specific factors that could explain differences in students' attitudes toward AI education. The observed UDS association should therefore not be interpreted as evidence of a substantive institutional effect and requires confirmation in further studies.

Academic level was not significantly associated with curriculum endorsement in either the bivariate or multivariable analyses. Although 68.9% of respondents believed that clinical practice increased their knowledge of AI, clinical practice exposure was not independently associated with curriculum endorsement after adjustment for the other variables included in the model. These findings should not be interpreted as evidence regarding the extent or quality of AI exposure during clinical training, as the study did not objectively assess AI-related content or experiences within clinical rotations.

The sensitivity analysis provided additional context for interpreting the multivariable findings. When students who responded “Not sure” to curriculum inclusion were excluded, a positive perception of AI's overall impact remained significantly associated with curriculum endorsement, suggesting that this association was relatively consistent across the two outcome definitions. In contrast, the gender and institution-specific associations changed when uncertain responses were excluded. These secondary findings should therefore be interpreted cautiously, as they appear to be sensitive to the classification of students who were uncertain about AI curriculum integration.

### 4.1 Implications for medical imaging education in Ghana

The high level of support for formal AI education observed in this study indicates substantial student receptiveness to the inclusion of AI education within undergraduate diagnostic medical imaging programmes in Ghana. This finding should not, however, be interpreted as evidence that students are ready for AI-enabled clinical practice or that specific AI knowledge deficits have been objectively established. The present study assessed awareness, self-reported exposure, attitudes, and curriculum preferences rather than objective AI knowledge or competency. Curriculum developers should therefore note that this study establishes student interest in AI education but does not assess students' current knowledge or readiness. Separate needs assessments using objective measures of knowledge and competency gaps would strengthen curriculum planning.

Subject to such needs assessment, potential curriculum content may include foundational concepts in artificial intelligence and machine learning, the role of training and validation data, and applications relevant to medical imaging, including image acquisition, reconstruction, image analysis, workflow support, and clinical decision support. Training may also address the limitations of AI systems, algorithmic bias, patient data protection, ethical considerations, accountability, and the continued role of professional judgement and human oversight.

Implementation should take account of differences in resources and technological capacity across universities. Rather than requiring the immediate introduction of a standalone advanced AI course, a phased approach may be more feasible. Foundational AI concepts could initially be incorporated into relevant existing courses, such as imaging informatics, digital image processing, research methods, and clinical imaging courses. Teaching could combine lectures with case-based discussions, demonstrations, simulation exercises, and supervised evaluation of AI-generated outputs. Where access to clinical AI systems is limited, educational software, open datasets, and simulation-based activities may provide introductory practical exposure without requiring substantial investment in commercial clinical systems.

Faculty capacity is also central to sustainable implementation. Lecturers and clinical educators would require opportunities to develop appropriate competencies in AI concepts, applications, limitations, and ethical issues. Interdisciplinary collaboration among medical imaging departments, medical physicists, radiologists, radiographers, computer scientists, and health informatics specialists could support teaching and reduce duplication of limited expertise. Collaboration among the participating universities may also facilitate the development of shared teaching resources and common minimum learning outcomes.

Resource constraints remain an important consideration, particularly because 72.3% of respondents endorsed the proposition that high cost could hinder AI implementation in Ghana. Curriculum planning should therefore distinguish between the resources required to teach fundamental AI concepts and those required for full clinical deployment of AI systems. Introductory AI education can begin without each institution acquiring advanced commercial AI platforms. A progressive model that begins with theoretical foundations, accessible computational resources, demonstrations, and simulated clinical applications may provide a more feasible pathway while institutional infrastructure and faculty expertise continue to develop.

### 4.2 Strengths and limitations

A major strength of this study is the inclusion of diagnostic medical imaging students from all five accredited public universities offering undergraduate training in Ghana. This provides broader institutional coverage than a single-centre study and permits examination of students' perceptions across different training settings. The study also extends previous descriptive assessments of AI awareness by examining factors associated with students’ endorsement of formal AI education.

Several limitations should nevertheless be considered. First, although the target sample was proportionately allocated across participating institutions, individual participants were recruited through convenience sampling using electronic student networks and WhatsApp platforms. Students with greater internet access, more frequent engagement with these platforms, or stronger interest in AI may therefore have been more likely to participate. Selection and non-response bias cannot be excluded, and the findings should not be interpreted as probability-based estimates of all diagnostic medical imaging students in Ghana.

Second, the study relied on self-reported awareness, familiarity, AI use, and perceptions. Self-reported familiarity with AI does not necessarily reflect objective knowledge or competency, and responses may have been affected by recall or social desirability bias. The study therefore cannot establish students' actual ability to understand, critically evaluate, or use AI systems in medical imaging practice.

Third, although the questionnaire was pilot-tested to establish clarity and comprehensibility before the main survey, comprehensive psychometric validation was not undertaken. The awareness and perspective domains demonstrated limited internal consistency, with Cronbach's alpha values of 0.46 and 0.57, respectively. These values fall below commonly accepted thresholds for reliable multi-item scales and therefore do not support interpretation of the respective domains as unified awareness or perspective constructs. The low internal consistency may reflect the heterogeneous content of the items, which assessed distinct perceived benefits, concerns, and implications of AI. For this reason, the items were analysed and interpreted individually rather than combined into composite scores. Nevertheless, the limited internal consistency and psychometric validation of the instrument restrict the strength of conclusions that can be drawn about broader latent constructs of AI awareness or attitudes.

Fourth, the cross-sectional design permits identification of statistical associations but cannot establish temporality or causality. For example, although a positive perception of AI was associated with curriculum endorsement, it cannot be determined whether favourable perceptions preceded support for AI education or whether students who already supported AI education were more likely to perceive AI positively.

Finally, the multivariable model demonstrated modest explanatory performance (McFadden's *R*^2^ = 0.099), suggesting that potentially important factors associated with curriculum endorsement were not captured. Variables such as previous formal AI training, objective digital literacy, access to computing resources, faculty exposure to AI, and institution-specific AI educational resources were not assessed. Future studies should incorporate objective measures of AI knowledge and competency, more extensively validated instruments, and additional educational and institutional factors. Qualitative or mixed-method approaches may also help explain why students support, oppose, or remain uncertain about formal AI education.

The closed-ended wording of several attitudinal items represents an additional measurement limitation. Some items were presented as declarative propositions concerning potentially adverse professional or implementation consequences of AI, including job insecurity, professional displacement, reduced professional knowledge, workload, and implementation cost. The direction and wording of such statements may have introduced framing or acquiescence effects. Because the study did not directly assess response-style bias or compare alternative item formulations, the presence or magnitude of these effects cannot be determined. Responses to these items should therefore be interpreted as endorsement or non-endorsement of prespecified propositions rather than as spontaneously expressed concerns or independently generated beliefs about AI.

Furthermore, because recruitment was conducted through electronic student networks without individualized tracking of survey invitations, the number of eligible students who received or viewed the invitation could not be established. A conventional response rate could therefore not be calculated, which limits assessment of potential non-response bias.

## 5 Conclusion

Diagnostic medical imaging students across the five participating universities in Ghana demonstrated high awareness of AI, substantial self-reported exposure to AI-enabled products or services, and generally positive attitudes toward its potential applications in medical imaging. Most respondents perceived potential benefits for diagnostic accuracy, clinical decision-making, and radiation dose optimisation, while 76.0% supported the inclusion of AI education in the medical imaging curriculum. Substantial proportions of respondents also endorsed prespecified propositions concerning potential professional displacement, machine-related errors, loss of professional knowledge, and implementation costs. These responses should be interpreted as attitudes toward the propositions presented rather than as spontaneously expressed concerns.

These findings demonstrate substantial student receptiveness to formal AI education and support consideration of its inclusion within undergraduate diagnostic medical imaging programmes in Ghana. However, they do not establish students' objective AI knowledge, competency, or readiness for clinical AI use. Potential curriculum content may include foundational AI concepts, relevant clinical applications, limitations of AI systems, ethical considerations, algorithmic bias, patient data protection, and appropriate human oversight. Practical approaches such as case-based learning, demonstrations, and simulation may complement theoretical instruction, particularly where access to clinical AI systems is limited.

Curriculum integration should take account of differences in institutional resources and faculty capacity and may be introduced progressively through relevant existing courses. Further research using objective measures of AI knowledge and competency, more extensively validated instruments, and broader sampling approaches is needed to better define students' educational needs and guide the development of AI education in diagnostic medical imaging programmes in Ghana.

## Data Availability

The original contributions presented in the study are included in the article/Supplementary Material, further inquiries can be directed to the corresponding author.

## References

[1] Hosny A Parmar C Quackenbush J Schwartz LH Aerts HJWL. Artificial intelligence in radiology. Nat Rev Cancer. (2018) 18(8):500–10. 10.1038/s41568-018-0016-5 29777175 PMC6268174

[2] Rajpurkar P Irvin J Ball RL Zhu K Yang B Mehta H. Deep learning for chest radiograph diagnosis: a retrospective comparison of the CheXNeXt algorithm to practicing radiologists. PLoS Med. (2018) 15(11):e1002686. 10.1371/journal.pmed.1002686 30457988 PMC6245676

[3] van De Venter R Skelton E Matthew J Woznitza N Tarroni G Hirani SP. Artificial intelligence education for radiographers, an evaluation of a UK postgraduate educational intervention using participatory action research: a pilot study. Insights Imaging. (2023) 14(1):25. 10.1186/s13244-023-01372-2 36735172 PMC9897152

[4] Antwi WK Akudjedu TN Botwe BO. Artificial intelligence in medical imaging practice in Africa: a qualitative content analysis study of radiographers’ perspectives. Insights Imaging. (2021) 12(1):80. 10.1186/s13244-021-01028-z 34132910 PMC8206887

[5] Botwe BO Antwi WK Arkoh S Akudjedu TN. Radiographers’ perspectives on the emerging integration of artificial intelligence into diagnostic imaging: the Ghana study. J Med Radiat Sci. (2021) 68(3):260–8. 10.1002/jmrs.460 33586361 PMC8424310

[6] Wuni AR Botwe BO Akudjedu TN. Impact of artificial intelligence on clinical radiography practice: futuristic prospects in a low resource setting. Radiography. (2021) 27:S69–73. 10.1016/j.radi.2021.07.021 34400083

[7] Adam AM. Sample size determination in survey research. J Sci Res Rep. (2020) 26:90–7. 10.9734/jsrr/2020/v26i530263

[8] Ampofo JW Emery CV Ofori IN. Assessing the level of understanding (knowledge) and awareness of diagnostic imaging students in Ghana on artificial intelligence and its applications in medical imaging. Radiol Res Pract. (2023) 2023:1–9. 10.1155/2023/4704342 PMC10287516 37362195

[9] Pinto dos Santos D Giese D Brodehl S Chon SH Staab W Kleinert R. Medical students’ attitude towards artificial intelligence: a multicentre survey. Eur Radiol. (2019) 29(4):1640–6. 10.1007/s00330-018-5601-1 29980928

[10] Sit C Srinivasan R Amlani A Muthuswamy K Azam A Monzon L. Attitudes and perceptions of UK medical students towards artificial intelligence and radiology: a multicentre survey. Insights Imaging. (2020) 11(1):14. 10.1186/s13244-019-0830-7 32025951 PMC7002761

[11] Edzie EKM Dzefi-Tettey K Asemah AR Brakohiapa EK Asiamah S Quarshie F. Perspectives of radiologists in Ghana about the emerging role of artificial intelligence in radiology. Heliyon. (2023) 9(5):e15558. 10.1016/j.heliyon.2023.e15558 37153404 PMC10160753

[12] Kelly CJ Karthikesalingam A Suleyman M Corrado G King D. Key challenges for delivering clinical impact with artificial intelligence. BMC Med. (2019) 17(1):195. 10.1186/s12916-019-1426-2 31665002 PMC6821018

[13] Masters K. Artificial intelligence in medical education. Med Teach. (2019) 41(9):976–80. 10.1080/0142159X.2019.1595557 31007106

